# Postoperative dry eye following femtosecond laser-assisted cataract surgery: insights and preventive strategies

**DOI:** 10.3389/fmed.2024.1443769

**Published:** 2024-11-18

**Authors:** Bin Lin, Dong-kan Li, Ling Zhang, Long-long Chen, Ying-ying Gao

**Affiliations:** ^1^School of Medicine, Xiamen Eye Center and Eye Institute of Xiamen University, Xiamen, China; ^2^Xiamen Clinical Research Center for Eye Diseases, Xiamen, Fujian, China; ^3^Xiamen Key Laboratory of Ophthalmology, Xiamen, Fujian, China; ^4^Fujian Key Laboratory of Corneal & Ocular Surface Diseases, Xiamen, Fujian, China; ^5^Xiamen Key Laboratory of Corneal & Ocular Surface Diseases, Xiamen, Fujian, China; ^6^Translational Medicine Institute of Xiamen Eye Center of Xiamen University, Xiamen, Fujian, China; ^7^Department of Ophthalmology, The Second Affiliated Hospital of Fujian Medical University, Quanzhou, Fujian, China

**Keywords:** femtosecond laser, cataract, dry eye, pathogenesis, patient interface

## Abstract

Postoperative dry eye is a common complication following femtosecond laser-assisted cataract surgery, and the patient interface (PI) used during the procedure may play a significant role in its occurrence. This study, utilizing a meticulous scientific search strategy, identified seven relevant articles through literature search engines. Most of these studies employed contact-type PI during surgeries, while one researcher used a non-contact PI. All studies assessed dry eye symptoms at various postoperative periods using metrics such as the Ocular Surface Disease Index (OSDI), tear Break-Up Time (BUT), Schirmer I test (SIt), and so on. However, the findings were inconsistent. On this basis, this comprehensive review delves into the potential impact of different patient interfaces on corneal nerve damage and conjunctival goblet cell injury, possibly contributing to an increased risk of postoperative dry eye. The review also explores various preventive and solution strategies, including improving PI design, reducing surgical time, and utilizing tear protective agents. The findings highlight the importance of optimizing the PI to minimize the risk of postoperative dry eye in femtosecond laser-assisted cataract surgery.

## 1 Introduction

Dry eye, a multifactorial disease affecting the ocular surface, has a global prevalence of 5 to 35% (1). Its complex causes and recurrent nature have intensified efforts toward better diagnosis and treatment. Ocular surgeries, notably cataract operations, can trigger or worsen dry eye symptoms, impacting patient satisfaction (2, 3). Due to its ultra-short pulses capable of releasing energy in an extremely brief duration and precisely cutting tissues (4), in recent years, Femtosecond Laser-Assisted Cataract Surgery (FLACS) has gradually become a popular new surgical method (5). Femtosecond laser technology provides highly accurate imaging of the anterior segment, enabling precise capsulorhexis, pre-chopping, clear corneal incision creation, and even limbal relaxing incisions to correct corneal astigmatism. This reduces the risk of capsular rupture during surgery, lowers the required phacoemulsification energy, and further diminishes corneal damage (6). Consequently, this approach not only significantly reduces the difficulty of the procedure but also maximally enhances visual outcomes. FLACS improves postoperative visual quality but does not simultaneously control the incidence of postoperative dry eye. Notably, compared to traditional cataract surgery, studies suggest that patients undergoing FLACS may be more prone to symptoms of dry eye (7). The exploration of dry eye pathogenesis following FLACS is crucial for developing effective prevention and treatment, thereby improving patients’ postoperative visual comfort and quality of life. Current research on this subject is sparse, necessitating a review of the potential mechanisms of dry eye post-FLACS.

## 2 Epidemiology

Cataract is one of the most common blinding eye diseases worldwide (8), accounting for over 50% of vision loss globally, including 33.4% of blindness and 18.4% of moderate to severe visual impairment (9). In recent years, FLACS has been accepted by more and more patients due to its several advantages, such as less ultrasound energy release and higher surgical success rates (10).

Data indicate that 68.9% of patients report a sensation of foreign body in the eye after FLACS, and 48.3% experience dryness of the eye postoperatively (11). In cases where patients with pre-existing dry eye were excluded before surgery, the incidence of newly diagnosed dry eye 1 week after FLACS could reach as high as 20.9%. This figure drops to 10.82% 1 month postoperatively, and by 3 months after surgery, 1.92% of patients still suffer from surgically induced dry eye (12). Patients with preoperative dry eye symptoms are at a higher likelihood of experiencing various degrees of symptom exacerbation after the surgery, clearly presenting a less than optimistic picture.

We employed a Boolean logic search strategy with a timeframe extending from the database’s inception to October 2023. The search strategy was as follows: (Femtosecond Laser-Assisted Cataract Surgery [Title/Abstract] OR FLACS [Title/Abstract]) AND (Dry Eye Syndrome [Title/Abstract] OR Dry Eye Disease [Title/Abstract] OR Keratoconjunctivitis Sicca [Title/Abstract] OR tear film [Title/Abstract] OR ocular surface [Title/Abstract] OR MGD [Title/Abstract] OR conjunctiva [Title/Abstract]) NOT (LASIK [Title/Abstract] AND SMILE [Title/Abstract]). The search engine used was PubMed, which yielded 11 relevant articles. Upon careful review, we identified that 1 article was a meta-analysis, 1 was focused on adjunctive dry eye medication treatment, 1 involved optical coherence tomography in cataract studies, 2 were clinical controlled trials mentioning postoperative dry eye but lacking specific quantitative data, 1 was a case report, and 1 involved related optical analysis. Consequently, 4 articles were selected. A similar search strategy was employed in China National Knowledge Infrastructure (CNKI) and Wanfang databases, resulting in 3 additional articles meeting the criteria.

We reviewed papers and found that only two studies (13, 14) indicated a reduction in dry eye symptoms after FLACS compared to conventional phacoemulsification surgery (CPS), while other retrieved articles all suggested an exacerbation of dry eye after FLACS (7, 11, 12, 15, 16), upon careful examination of these two studies, we found that FLACS was associated with varying degrees of worsening dry eye symptoms compared to preoperative levels, albeit not as severe as in the CPS group. This may be related to the surgical techniques used during CPS and the stimulation of the ocular surface during the femtosecond laser procedure. In particular, the stimulation of the ocular surface by the patient interface (PI) during FLACS may promote increased secretion of reflex tears postoperatively (17), resulting in better performance in certain dry eye evaluation indices compared to CPS. Additionally, the surgeon’s operative habits are an important factor that cannot be ignored. Different surgical techniques may have a significant impact on the extent of ocular surface damage, thereby affecting the development of dry eye symptoms. The current status of related research both domestically and internationally is detailed in Table 1.

**Table 1:** Information and characteristics of international relevant research status.

Author	Year	Femtosecond laser machine	n*	Follow-up duration	Design of experiments	Outcomes	Dry eye markers
Xu et al. (11)	2021	LenSX	416 eyes	3 months	It is a Retrospective cohort study. Single-factor analysis and multivariable logistic regression were utilized to investigate potential risk factors for dry eye following FLACS.	Factors identified as significant risks included female gender, history of alcohol consumption, type 2 diabetes, meibomian gland dysfunction, previous ocular surgeries, high cumulative released energy, and prolonged duration of vacuum suction.	OSDI TMH SIt FS BUT
Marc Schargus et al. (16)	2020	Catalys	17 eyes vs. 17 eyes	3 months	It is a Prospective randomized controlled trial. A sequential cohort was randomly assigned to undergo either FLACS or CPS. Evaluations of the dry eye were conducted, including measurements of tear film osmolarity, SIt, MMP-9, corneal sensitivity, and so on.	At 1 and 3 months post-treatment, no significant differences were found between groups in tear film osmolarity, SIt, or MMP-9 levels.	MMP-9 SIt TOP
Ju et al. (10)	2019	LensSX	38 eyes	3 months	It is a Before-and-after control trial. Detailed recording of femtosecond laser parameter settings, including not only the position and size of the corneal incision but also capsulotomy size and laser energy level.	Corneal fluorescein staining scores significantly improved post-surgery (*p* < 0.05). OSDI scores spiked post-surgery (*p* < 0.05), remaining high at 3 months versus baseline. The other indicators of dry eye worsened early and then gradually improved over a 3-month follow-up period.	OSDI TMH SIt BUT FS SS score
Shao et al. (15)	2018	LensSX	150 eyes vs. 150 eyes	3 months	It is a Randomized controlled trial. Preoperatively excluded the patients with systemic diseases potentially affecting dry eye, local ocular inflammation, and a history of related local medication use.	Dry eye index increased more significantly in the FLACS group than in the CPS group 1 day and 1 week after surgery, but there was no statistically significant difference at 3 months after surgery.	OSDI TMH SIt FS BUT
Chen et al. (12)	2018	N/A	86 eyes vs. 75 eyes	3 months	It is a Prospective cohort study. The study was grouped according to different surgical procedures. However, it did not provide the femtosecond laser model and related parameter settings, lacking baseline data for research.	There was no statistically significant difference between the two groups pre-operation and 3 months after surgery, but there was a statistically significant difference at 1 week and 1 month after surgery (*p* < 0.05). FLACS had a lower effect on tear quality than CPS.	SIt FS BUT
Zhou et al. (13)	2018	LenSX	26 eyes vs. 27 eyes	3 months	It is a Prospective cohort study. This study provided a more comprehensive comparison of baseline characteristics of the population, including age, gender, operative eye, and cataract nucleus grading, among others.	Post-operation, both groups’ SIt, and subjective dry eye scores dipped then rose, showing no statistical difference. By 3 months, metrics nearly matched pre-op levels, but CPS’s 3-month subjective dry eye score remained higher than pre-op, with statistical significance (*p* < 0.05).	MCW score SIt BUT CS
Yu et al. (14)	2015	LenSX	73 eyes vs. 64 eyes	1 month	It is a Prospective, non-randomized controlled study. Excluded the patients who had used artificial tears and NSAIDs within the past months. Dry-eye markers including the OSDI, subjective symptom questionnaire, tear-film assessment, SIt, and fluorescein staining.	The study found a significant worsening in dry eye after both groups. However, the symptoms of dry eye were more pronounced in the FLACS group at 1 week and 1 month postoperatively (*p* < 0.05).	OSDI TMH SIt BUT FS

*Sample size for the before-and-after control trial of FLACS is expressed as N, and for the FLACS vs. CPS trial, sample size is expressed as femtosecond group N1 vs. phacoemulsification group N2.

TOP, Tear osmotic pressure; OSDI, Ocular Surface Disease Index; TMH, Tear Meniscus Height; SIt, Schirmer I test; BUT, tear Break-Up Time; FS, fluorescence staining, SS score-Subjective symptom score, MCW score-Mcmonnies CW questionnaire score; CS, corneal sensation; MMP-9, Matrix Metalloproteinase-9.

## 3 Mechanisms of postoperative dry eye

### 3.1 Common mechanisms of CPS and FLACS

Postoperative dry eye can occur to varying degrees after CPS and FLACS, with several common mechanisms identified in the development of dry eye following both procedures. Past research results suggest there are several reasons for dry eye after surgery. First, the creation of corneal incisions, whether using a scalpel or femtosecond laser, leads to the transection of corneal nerves to varying extents. This delays corneal wound healing, reduces corneal sensitivity, and impedes the tear secretion reflex among other phenomena (18). Second, to maintain ocular surface moisture and corneal transparency during surgery, balanced salt solutions are often used to repeatedly rinse the ocular surface, which may cause damage to the corneal epithelium and conjunctival goblet cells (19, 20). Third, an increased postoperative inflammatory response, leading to the recruitment of neutrophils and macrophages as well as the production of free radicals, proteolytic enzymes, and cyclooxygenase, is also considered a key factor in the development of dry eye (21). Fourth, studies have shown that more than 70% of patients experience meibomian gland orifice blockage and lid margin hyperemia 1 month after cataract surgery, leading to a significant reduction in the thickness of the tear film lipid layer and exacerbation of dry eye symptoms (22). This may be related to postoperative inflammatory responses, reduced blink frequency, and frequent use of eye drops. Fifth, prolonged exposure to microscope light is also associated with a shortened tear film break-up time and the exacerbation of dry eye symptoms in the short term (23). Sixth, the use of topical anesthetic agents during surgery can damage structures such as corneal epithelial microvilli, further affecting the normal adhesion of mucins and resulting in decreased stability of the tear film (24).

### 3.2 Mechanisms of postoperative dry eye after FLACS

#### 3.2.1 More severe postoperative inflammatory response

The PI causes additional damage to ocular surface tissues compared to CPS (16), and FLACS is associated with a more severe inflammatory response in the anterior segment of the eye (25). This alters the ocular surface microenvironment, leading to changes in the concentration of tear cytokines (26). Patients with dry eye often have higher levels of pro-inflammatory cytokines in their tears (27), such as Interleukin-1β (IL-1β), Interleukin-6 (IL-6), Interleukin-8 (IL-8), and MMP-9 (28). Studies have shown that the concentrations of pro-inflammatory cytokines like IL-6 and IL-8 in the eye post-FLACS are significantly higher compared to CPS (29). This exacerbates ocular surface inflammation, affects tear film quality (30), and leads to ocular surface dryness. The dry, oxidative, and hyperosmolar ocular surface environment activates cellular signaling pathways on the ocular surface, such as the Mitogen-Activated Protein Kinases (MAPK) signaling pathway and the Nuclear Factor kB (NF-kB) signaling pathway (31, 32), further stimulating the production of corresponding inflammatory cytokines in a vicious cycle.

#### 3.2.2 Additional damage to corneal

Due to the high precision of femtosecond lasers, the corneal incisions they create are well-cemented. Surgeons often need to employ specific techniques to bluntly separate the completed incisions for further surgical procedures (33, 34). However, this process can be challenging, as unlike the sharp, disposable scalpel used in CPS, which typically completes the incision in one go, the corneal incisions made by femtosecond lasers may sometimes be incomplete or discontinuous and cannot simply be resolved by blunt separation, and may still require the use of a scalpel (35), potentially causing reinjury to the cornea. Additionally, it is worth noting that the precision of the corneal incisions largely depends on the stability of PI, which is crucial for the accurate focus of the femtosecond laser on the cornea. So unstable PI suction could cause unnecessary damage to the cornea (36), affecting the healing of the incision and the overall quality.

#### 3.2.3 Damage to conjunctival goblet cells

**Figure 1:**
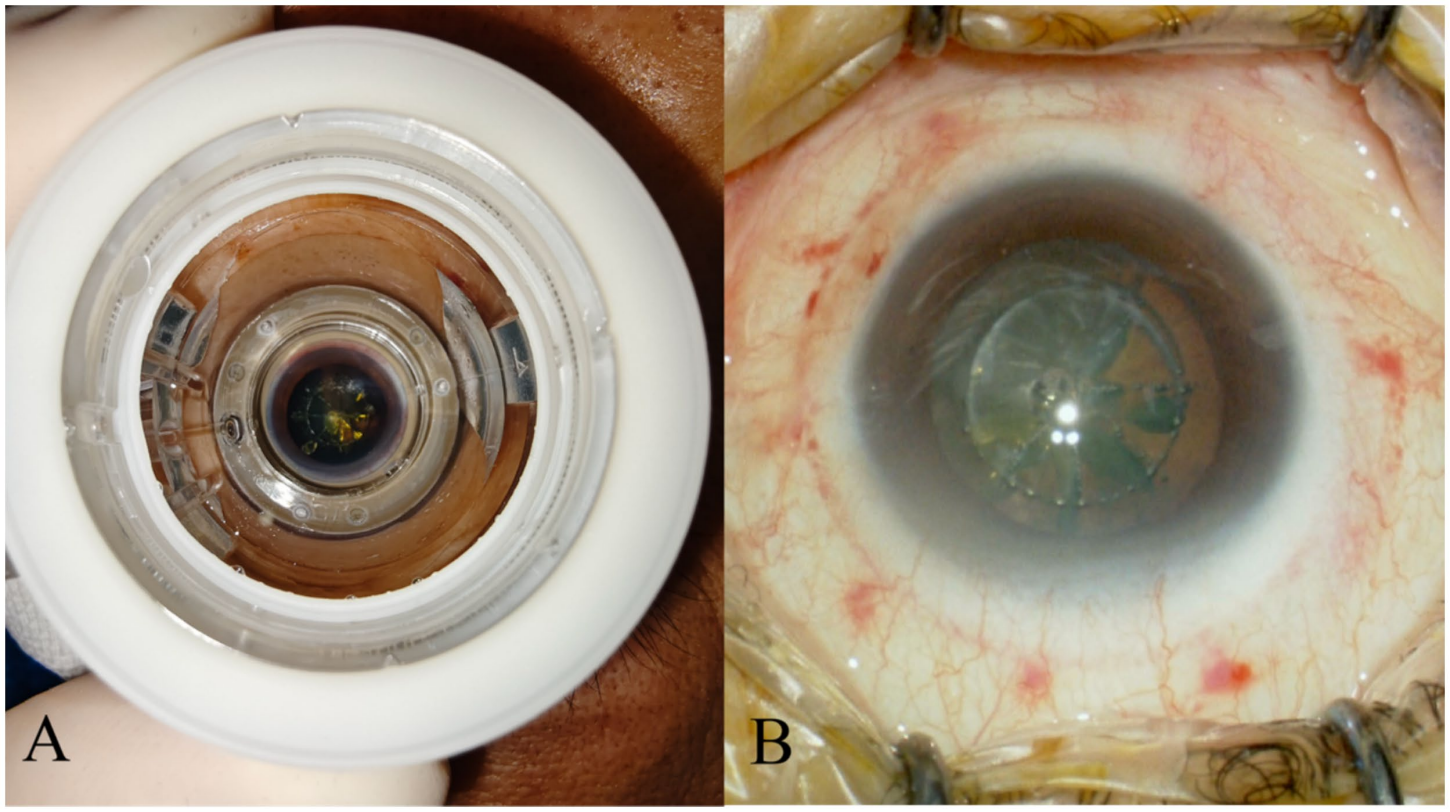
PI left obvious marks on conjunctival tissue. **(A)** Illustrates the condition when PI is adsorbed onto the patient’s ocular surface. **(B)** Shows the residual traces of PI on the ocular surface tissues after the negative pressure is released.

Goblet cells are responsible for producing the mucin component of the tear film, which helps the tear film to stably adhere to the ocular surface. However, both contact and non-contact femtosecond laser systems require the application of PI vacuum fixation on the patient’s ocular surface. The negative pressure attraction and compression of PI on the conjunctival tissue can cause partial apoptosis and decreased density of conjunctival goblet cells (37), with contact PI theoretically causing more severe damage. Which is similar to the pathological changes in postoperative dry eye caused by femtosecond laser-assisted *in situ* keratomileusis (LASIK) (38). Shao et al. also confirmed through research that the conjunctival damage caused by PI during FLACS is greater than that in the CPS group (16). In addition to changes in the number of goblet cells, Chao et al. found that the mucin secretion function of goblet cells was also affected (39), leading to mucin-deficient dry eye. The impact of PI vacuum attraction on the conjunctiva is detailed in Figure 1.

#### 3.2.4 Compression effect of PI on the ocular surface

The tear film is fundamental to maintaining the normal structure and function of the ocular surface epithelium, as it moisturizes and protects the cornea and conjunctival epithelium (16). When the integrity of the tear film layers is compromised, tear film break-up occurs, leading to increased tear evaporation. During the femtosecond laser procedure, PI is applied to the patient’s ocular surface. The mechanical compression effect results in a decrease in the regularity of the postoperative ocular surface. This causes discordant interaction between the posterior edge of the eyelids and the corneal surface during blinking, which is not conducive to the tear film uniformly and smoothly covering the ocular surface (11), affecting tear film stability and thereby shortening the tear film break-up time.

#### 3.2.5 Damage to the ocular surface nerves by PI

The ocular surface tissue involves multiple nerves and sensory systems (40), for example: the corneal nerves, which are a major component of the ocular surface nerves responsible for transmitting tactile and pain sensations, and are crucial for maintaining blinking and tear reflexes (41). During FLACS, both the non-contact PI’s negative pressure suction on the ocular surface and the contact PI’s compression (42–44), cause damage to various ocular surface nerves. Pathological changes in corneal nerves are a primary cause of corneal neuropathic pain. And damaged corneal nerves also cause neuroinflammation and sensitization, thereby forming a vicious cycle (45).

#### 3.2.6 Additional surgical time and perioperative medication

**Figure 2:**
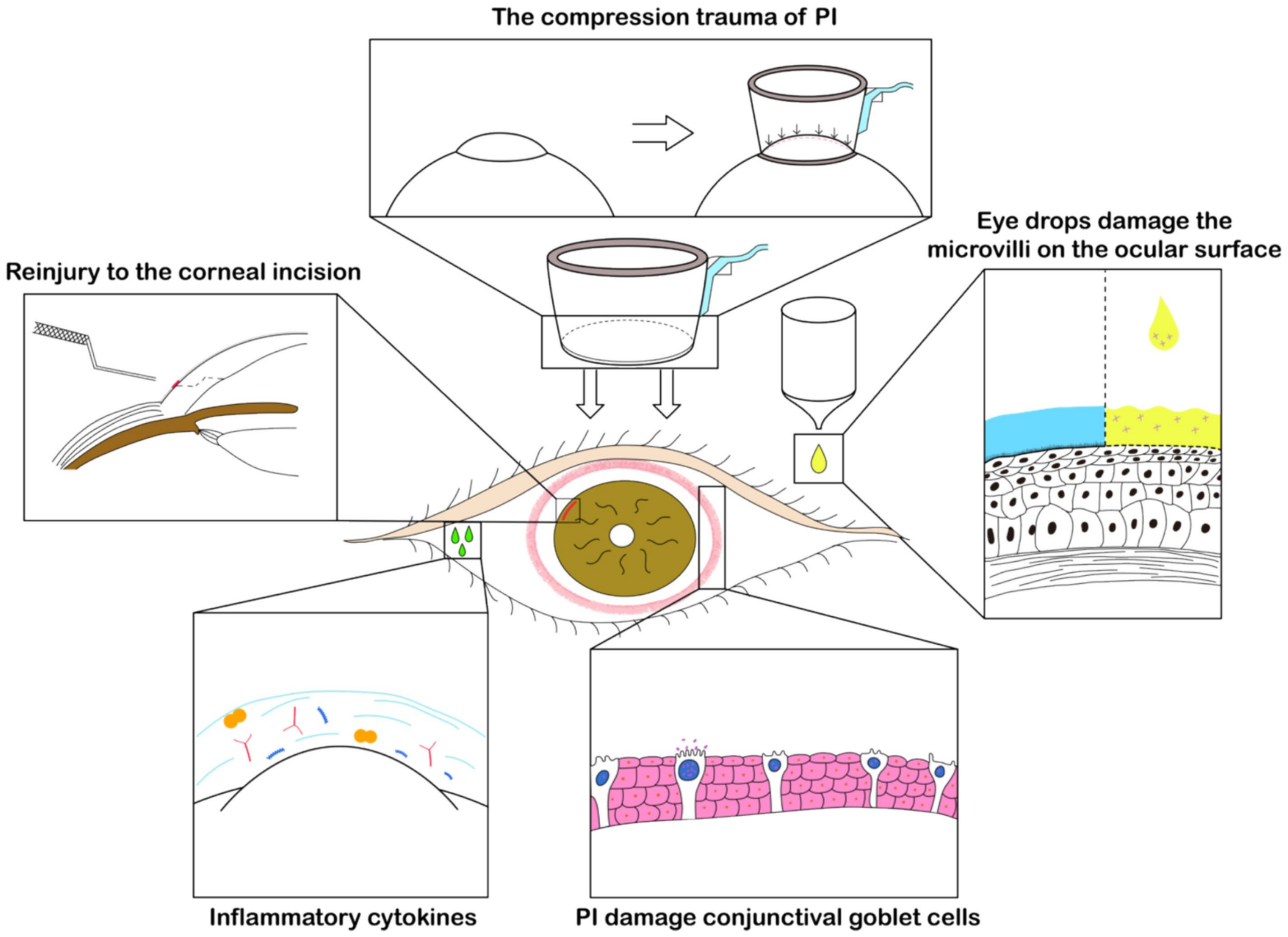
Schematic diagram of FLACS effect on dry eye. The negative pressure suction by the PI left noticeable marks on the conjunctiva, evidencing pressure-induced trauma. This also damages the conjunctival goblet cells, affecting the concentration of inflammatory cytokines on the ocular surface. The use of a specific corneal incision separator may also cause reinjury to the cornea. Moreover, the perioperative increase in the use of eye drops for FLACS such as non-steroidal anti-inflammatory drugs contributes to damage to the eye’s microvilli, playing a significant role in postoperative dry eye.

Due to the need of operations such as creating clear corneal incisions, capsulorhexis, and pre-chopping of the nucleus on the femtosecond laser platform, there is an additional increase in the time the patient’s ocular surface is exposed and the use of topical anesthetic drugs (46). Studies indicate that the longer the ocular surface tissue is exposed during surgery, the more likely it is to cause damage to the microvilli structure of ocular surface cells and a postoperative decrease in goblet cell density (20). Pupil constriction after femtosecond laser operation is a common complication of FLACS. Some specialists suggest “To reduce the occurrence of pupil constriction after femtosecond laser operation, the preoperative use of non-steroidal anti-inflammatory drugs and increased dosage of mydriatic eye drops is recommended (47).” However, excessive use of non-steroidal anti-inflammatory drugs may lead to slower corneal epithelial healing (48). Despite the well-documented potential to exacerbate dry eye symptoms, the inclusion of the preservative benzalkonium chloride (BAC) in ophthalmic solutions is common practice (49). Compared to traditional phacoemulsification cataract surgery, the use of additional medications in FLACS inevitably increases exposure to preservatives such as BAC. The preservatives in the drugs also cause damage to the ocular surface (50) and reduce the density of conjunctival goblet cells. A schematic diagram illustrating the specific mechanism, created by the authors, is presented in Figure 2.

## 4 Prevention and treatment measures

### 4.1 Routine perioperative prevention and treatment of dry eye

It is important to assess dry eye symptoms before surgery. Gupta et al. found that about 80% of patients had abnormal dry eye test results before cataract surgery (51). Clinicians should conduct personalized risk assessments before surgery (52) and, based on the patient’s condition, use artificial tears or perform meibomian gland treatment to relieve dry eye symptoms.

During surgery, to reduce the damage caused by the irrigation of balanced salt solution on the ocular surface, it is recommended to use corneal protectants during surgery (53), or apply a diluted viscoelastic agent to the corneal surface in a certain ratio. This not only effectively maintains corneal moisture but also downregulates the expression of inflammatory factors, further promoting the repair of intraoperative ocular surface damage (54). After surgery, besides routine anti-inflammatory and artificial tear treatments, Intense Pulsed Light (IPL) therapy may be appropriately applied for patients at high risk of dry eye (55). Meanwhile, a corneal bandage lens can reduce mechanical damage to the ocular surface tissues by the eyelid margin, and diminish corneal neuropathy caused by eyelid-related factors (56). It is also suitable for people at high risk of dry eye.

### 4.2 Targeted prevention and treatment during the perioperative period of FLACS

#### 4.2.1 Femtosecond laser equipment and parameter adjustment

The effectiveness and safety of FLACS are closely related to the settings of femtosecond laser parameters. Surgeons need to finely adjust these parameters according to the individual differences and surgical needs of patients. For patients at risk of dry eye, it may be necessary for the doctor to further reduce the laser’s energy and duration of action to minimize potential damage to the ocular surface tissues (57), reducing the inflammatory response after femtosecond laser operation. Additionally, doctors can more specifically select femtosecond laser equipment and techniques based on the patient’s specific conditions (58). For example, for patients with small eyeballs, due to the greater curvature of the cornea, the use of a contact femtosecond laser device’s PI can cause significant compression on the limbal conjunctival tissue. In such cases, besides using a smaller size of PI model, it is also possible to choose a non-contact femtosecond laser device, if available, to avoid the generation of compression on ocular surface tissues.

#### 4.2.2 Targeted supplementation with artificial tears

To date, in the development of dry eye science, artificial tears that have been developed can simulate one or several components of the tear film, targeting the mucin layer, aqueous layer, and lipid layer for supplementation (59). Preservative-free artificial tears can be used multiple times throughout the day, and it is recommended for patients with dry eye after FLACS to prioritize the use of artificial tears for replacement therapy (60, 61). Using oily artificial tears to alleviate tear film kinetics abnormalities and lipid abnormality type dry eye caused by conjunctival laxity and meibomian gland dysfunction after FLACS can effectively relieve ocular discomfort (62).

#### 4.2.3 Mucin secretagogue treatment

The mucin layer is an essential component of the tear film, with functions that include providing lubrication to the ocular surface, facilitating tear distribution, maintaining tear film stability, and aiding in ocular surface repair. Currently, mucin secretagogue medications primarily include Diquafosol sodium eye drops (63) and Rebamipide (64). Through the analysis of the mechanisms behind post-FLACS dry eye, it is known that dry eye following FLACS is significantly related to the destruction of the mucin layer, therefore, Diquafosol sodium eye drops and Rebamipide can produce beneficial effects (65).

#### 4.2.4 Promotive repair treatment

Due to the possibility of more severe conjunctival epithelial damage and ocular surface nerve injury after FLACS, in addition to artificial tears, postoperative local promotive repair treatments can be administered, such as human epidermal growth factor eye drops, deproteinized calf blood extract eye drops, autologous serum, and so on. Human autologous serum contains components such as nerve growth factor, epidermal growth factor, and fibronectin, which help in the regeneration of nerves and epithelial cells. Thus, for patients with severe dry eye or those who do not respond to artificial tears treatment, the use of autologous serum can be considered (66). It is necessary to pay attention to the storage environment of autologous serum and avoid contamination (67).

#### 4.2.5 Anti-inflammatory treatment

Post-FLACS dry eye is often closely associated with inflammatory responses. The use of low-dose corticosteroid anti-inflammatory drugs can help alleviate local tissue inflammation and stabilize the tear film, which is of significant importance for the stability of the ocular surface post-FLACS (68). Beyond that, cyclosporine A, as an immunosuppressive agent, possesses unique capabilities to improve goblet cell density and anti-apoptotic properties (69). Research has confirmed that cyclosporine A significantly improves dry eye induced by cataract surgery (70). Therefore, the use of corticosteroid anti-inflammatory drugs and immunosuppressive medications is critically important for managing post-FLACS dry eye and maintaining the stability of the ocular surface.

## 5 Conclusion and prospects

The advent of FLACS undoubtedly represents a significant technological breakthrough in the field of ophthalmology (71). At the same time, we have also focused on the occurrence of postoperative dry eye. Currently, the clinical treatment of FLACS-induced dry eye often involves only symptomatic treatment with artificial tears to alleviate dry eye symptoms and signs, without precise etiological treatment. This is also the reason why some clinical patients have recurrent dry eye symptoms and prolonged illness. Personalized treatment of dry eye is a major trend at present. Only by gaining a deeper understanding of the various impacts that FLACS may have on the eye can we develop more effective prevention and treatment.

Through the discussion in this article, we have found that post-FLACS dry eye is related to multiple factors, such as changes in tear cytokines, corneal damage, inflammatory responses, and the pressure exerted by the PI on the eyelids and ocular surface tissues. Therefore, before and after surgery, we need to take a series of targeted preventive and therapeutic measures, such as preoperative assessment of the patient’s dry eye symptoms, optimization of femtosecond laser parameters, anti-inflammatory treatment, targeted supplementation of artificial tears, and promotion of mucin secretion, to reduce the risk of postoperative dry eye and alleviate postoperative dry eye symptoms. At the same time, we found that the damage caused by the PI to the ocular surface tissues cannot be ignored. Jonathan et al. have previously compared the safety and effectiveness of contact and non-contact PIs, finding that reducing the contact area between the PI and the ocular surface not only reduces damage to the ocular surface but also improves the efficacy of the femtosecond laser (42). We believe this could be a direction for future innovation and development of PIs, namely, achieving the same or even better suction effects with less contact with the ocular surface or softer and more comfortable contact materials, which will be beneficial for the repair of postoperative ocular surface tissues. Of course, this requires further clinical trials for validation.
